# Effects of Cement Dosage, Curing Time, and Water Dosage on the Strength of Cement-Stabilized Aeolian Sand Based on Macroscopic and Microscopic Tests

**DOI:** 10.3390/ma17163946

**Published:** 2024-08-08

**Authors:** Heng Yang, Zengzhen Qian, Bing Yue, Zilu Xie

**Affiliations:** 1 School of Engineering and Technology, China University of Geosciences, Beijing 100083, China; 2102220105@email.cugb.edu.cn (H.Y.); yue@email.cugb.edu.cn (B.Y.); a894932371@163.com (Z.X.); 2 Key Laboratory on Deep Geo-Drilling Technology, Ministry of Natural Resources, Beijing 100083, China

**Keywords:** aeolian sand, cement-stabilized aeolian sand, orthogonal experiments, unconfined compressive strength, microscopic mechanism

## Abstract

Aeolian sand is distributed worldwide, exhibiting poor grading, low cohesion, and loose structure. Infrastructure construction in desert areas sometimes requires stabilization of the sand, with cement as the primary curing agent. This study first employed orthogonal experiments to evaluate critical factors, e.g., curing time, cement dosage, and water dosage, affecting the unconfined compressive strength (UCS) of the aeolian sand stabilized with cement (ASC). Each of the aforementioned factors were set at five levels, namely curing time (7, 14, 28, 60, and 90 days), cement dosage (3%, 5%, 7%, 9%, and 11%), and water dosage (3%, 6%, 9%, 12%, and 15%), respectively. The water and cement dosages were percentages of the mass of the natural aeolian sand. The results indicated that the sensitivity of the influencing factors on the UCS of ASC was cement dosage, curing time, and water dosage in descending order. The UCS of ASC positively correlated with curing time and cement dosage, while it first increased and then decreased with the water dosage increase. The optimal conditions were 90 days’ curing time, 11% cement dosage, and 9% water dosage. The microscopic analyses of ASC using optical microscopy, scanning electron microscopy (SEM), and X-ray diffraction (XRD) revealed that hydration products enhanced strength by bonding loose particles and filling pores, thereby improving compaction. The quantity and compactness of hydration products in the aeolian–cement reaction system increased with the increases in cement dosage and curing time, and low water dosage inhibited the hydration reaction. This study can provide insights into the stabilization mechanism of aeolian sand, aiding infrastructure development in desert regions.

## 1. Introduction

Global desert distribution is extensive and diverse, and is primarily concentrated in the tropical regions between the Tropic of Cancer and the Tropic of Capricorn and in specific cold climate zones. According to the United Nations Environment Programme, approximately one-third of the Earth’s land area is covered by deserts. Major deserts include the Sahara Desert, the Arabian Desert, the Gobi Desert, and the Victoria Desert (in Australia) [1,2]. Desertification in China is predominantly concentrated in the northwestern and northern regions of the country, encompassing a total area of approximately 186,300 km^2^. Notable examples include the Taklamakan Desert, the Mu Us Desert, and the Kubuqi Desert [3,4,5,6]. Aeolian sand, prevalent in these desert regions, is a very peculiar material characterized by loose aggregation and uniform particle size distribution, with an inherent lack of cohesion. Therefore, before aeolian sand can be used as a subgrade for construction structures, it must undergo stabilization to ensure its integrity and stability [7,8]. The unique properties of aeolian sand pose both challenges and opportunities for stabilization. To enhance aeolian sand strength, physical methods like reinforcement, chemical methods, such as cement and alkali-activated material (AAM) stabilization, and biological methods, like microbially induced calcite precipitation (MICP), can be used [9,10,11]. Physical methods are relatively costly and the high temperature and arid environment of the desert inhibits microbial activity, resulting in MICP not achieving optimal stabilization effects. Therefore, chemical methods are more suitable for stabilizing aeolian sand in desert environments. AAM has several distinct advantages, such as environmental friendliness, durability, and cost-effectiveness, as it typically uses waste or industrial by-products as raw materials, reducing environmental pollution and lowering production costs [12]. However, there are also challenges and limitations associated with AAM technology. For example, its standardization and range of applications may be limited due to a lack of established guidelines [13], and AAM may not achieve early strength development as quickly as traditional cement, requiring a longer time to reach design strength [14]. This delayed strength development can be a significant drawback in projects where early strength is crucial. Compared with AAM, Portland cement excels in early strength development, reaching design strength in a shorter period and meeting the requirements for early strength in engineering projects [15]; it is also simpler to handle and apply.

Therefore, using Portland cement to improve the strength of aeolian sand remains a considerable method in practical conditions. Studies have emphasized the influence of cement and water content on the unconfined compressive strength (UCS) of cemented specimens. Mixing aeolian sand with cement has been found to offer adequate bearing capacity for low- to moderate-height buildings and improve the compaction and load-bearing capacity of aeolian sand. The influence of curing time, cement dosage, and water dosage on the mechanical properties of aeolian sand stabilized with cement (ASC) has also been investigated [16,17,18,19,20,21,22].

Despite extensive research on the stabilization of aeolian sand using cement, most previous studies have focused on examining two of the three factors (curing time, cement dosage, or water dosage). There remain gaps in understanding the optimal combination of curing time, cement dosage, and water dosage for maximizing the stabilization effect. To determine the impact of these three parameters and the optimal combination, a comprehensive analysis of all factor and level combinations is necessary. Traditional testing approaches would require a prohibitively large number of experiments. Thus, this study employs an orthogonal experimental design (a widely used multifactor experimental method that reduces the number of specimens while ensuring the accuracy of results).

Orthogonal experimental design allows for an efficient and systematic investigation of the effects of curing time, cement dosage, and water dosage on the UCS of ASC. The curing times selected for this study (7, 14, 28, 60, and 90 days) encompass the typical range used in engineering conditions, allowing for an assessment of both short-term and long-term strength development. The cement dosages (3%, 5%, 7%, 9%, and 11%) and water dosages (3%, 6%, 9%, 12%, and 15%) were chosen based on their relevance to practical applications and an existing study [23].

In addition to macroscopic strength measurements, this study employs optical microscopy, scanning electron microscopy (SEM), and X-ray diffraction (XRD) to explore the microstructural changes in ASC [8,24,25]. These techniques provide detailed insights into the bonding mechanisms, pore structure, and mineralogical transformations that occur during stabilization. The microscopic analysis elucidates how hydration products contribute to the increased mechanical properties of the ASC.

**Figure 1:**
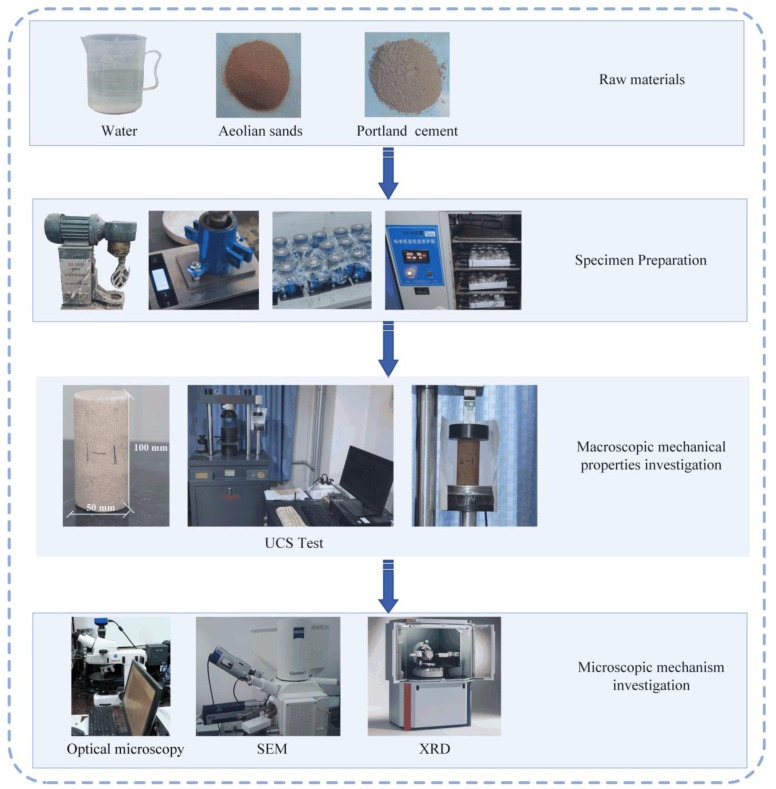
Experimental flow chart.

The specific research process of this paper is as follows: Initially, a series of specimens are prepared and cured according to the principles of orthogonal experimental design. Subsequently, these specimens undergo unconfined compression tests to measure their UCS, a critical indicator for assessing the macro-mechanical properties of ASC. Following this, the test data are subjected to an analysis to explore the specific effects of curing time, cement dosage, and water dosage on UCS, and to determine the optimal parameter combination design. Finally, the post-compression specimens are subjected to microstructural analysis using SEM and XRD to reveal the mechanism of cement action during the solidification of aeolian sand. The findings from the micro tests elucidate the changes in UCS observed in the macro-mechanical tests, and the correlation between macroscopic mechanical properties and microscopic changes has been established. The experimental flow chart can be seen in Figure 1.

The novel contributions of this research lie in its integrated approach, combining macroscopic and microscopic analyses to provide a holistic understanding of the stabilization mechanism of ASC. This study not only clarifies the influence of key factors on UCS but also provides practical recommendations for optimizing the stabilization process. The findings have significant implications for guiding infrastructure construction in desert areas, ensuring the durability and safety of engineering projects, and ultimately promoting economic and social development in these regions. By addressing the identified knowledge gaps and offering innovative insights, this research contributes to the advancement of techniques for stabilizing aeolian sand, making it a valuable resource for both academic and practical applications.

## 2. Materials and Methods

### 2.1. Materials

#### 2.1.1. Aeolian Sand

Aeolian sand used in this study was obtained from the Taklamakan Desert, Xinjiang, China. According to the Chinese national standard for geotechnical testing method (GB/T 50123-2019) [26], the natural sand moisture content was measured by the drying method. The nature porosity was measured by the irrigation method according to the Chinese transportation industry standard (JTGF10-006-2014) [27]. The moisture dosage, nature density, and natural porosity of aeolian sand were 0.54%, 1.38 g/cm^3^, and 51%, respectively. The results are listed in Table 1.

**Figure 2:**
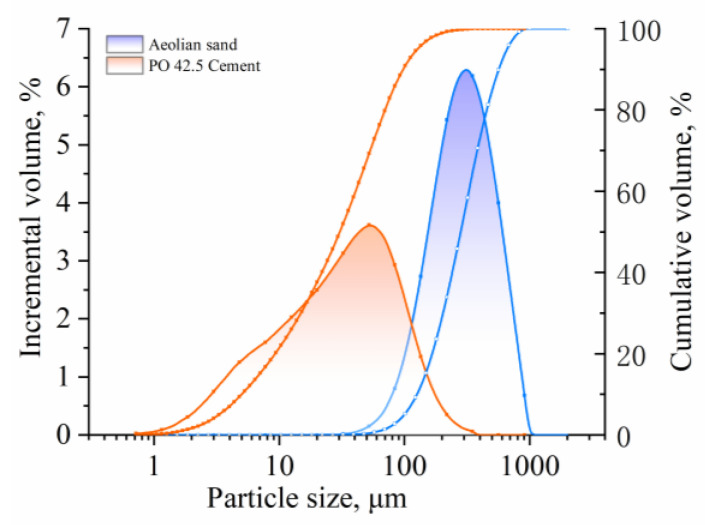
Particle size distributions of aeolian sand and cement.

The particle size distribution of aeolian sand was determined via a laser particle size analyzer (BT-2000, Better Company, Dandong, China). It can be seen from Figure 2 that the main particle sizes of aeolian sand ranged from 100 to 600 μm. The effective particle size was *d*_10_ = 131.95 μm, the median particle size was *d*_30_ = 210.22 μm, and the limited particle size was *d*_60_ = 345.58 μm. The coefficient of uniformity (*C*_u_) and coefficient of curvature (*C*_c_) of aeolian sand were 2.62 and 0.97, respectively, and these two values were similar to previous research [28]. Therefore, its natural gradation was discontinuous. The characteristic indexes of aeolian sand are also summarized in Table 1.

The chemical composition of the aeolian sand was analyzed by X-ray fluorescence (XRF) (XRF-1800, Shimadzu, Tokyo, Japan), which is given in Table 2. The main chemical components of aeolian sand were SiO_2_, Al_2_O_3_, and CaO. The mineral composition of the aeolian sand was determined using XRD, with the main mineral components and their proportions shown in Table 3.

**Figure 3:**
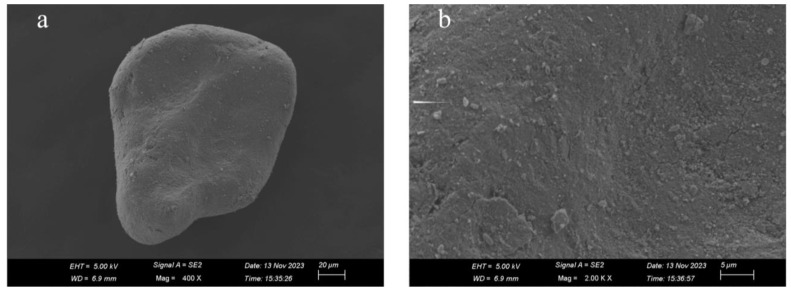
Microscopic morphology of aeolian sand particle. (**a**) Magnified 400×; (**b**) magnified 2000×.

In this study, a scanning electron microscope (Zeiss Sigma 500 field emission, Carl Zeiss, Oberkochen, Germany) was utilized to conduct a detailed observation and analysis of the micro-structure of aeolian sand [20,29]. As can be seen in Figure 3, Aeolian sand particles exhibited a near-spherical shape with no discernible edges and a relatively good degree of rounding. Moreover, the surface of the particles exhibited distinct signs of wear and cracking. The morphological characteristics of aeolian sand were attributed to the prolonged rolling, scouring, and friction during the transportation process.

#### 2.1.2. Cement

The cement used in this study was ordinary Portland cement (OPC), which was P.O 42.5 produced by China United Cement Corporation (Beijing, China). The test methods of OPC were similar to those of aeolian sand. The particle size distribution is shown in Figure 2 and the physical properties are shown in Table 1. The chemical composition of OPC was analyzed using XRF, with the main chemical components being calcium, silicon, and aluminum, as shown in Table 2.

### 2.2. Methods

#### 2.2.1. Orthogonal Experimental Design

Orthogonal experimental design is a widely used multifactor experimental method based on an orthogonal array, and the results of orthogonal experimental design can meet the requirements of a full factorial test [30,31,32]. The main purpose of this work is to study the effects of the three factors (Factor A: curing time, Factor B: cement dosage, and Factor C: water dosage), and the different factors’ combinations of collocation on the effects of UCS. Five different levels of curing times (A) were 7, 14, 28, 60, and 90 days, respectively. Five levels of cement dosage (B) were set as 3%, 6%, 9%, 12%, and 15%. The water dosage (C) was set as 3%, 5%, 7%, 9%, and 11%. Factors and levels can be seen in Table 4. The cement dosages and water dosages were percentages of the mass of natural aeolian sand. The calculations of cement dosage and water dosage are shown in Equations (1) and (2):

(1) $$cement\;dosage=\frac{m_{c}}{m_{s}}\times 100\%$$

(2) $$water\;dosage=\frac{m_{w}}{m_{s}}\times 100\%$$

where *m*_c_ represents the mass of cement; *m*_w_ represents the mass of added water; *m_s_* represents the mass of natural aeolian sand.

For orthogonal table L_n_ (k^m^), L represents the name of the orthogonal table, n is the number of experiments, k is the number of levels for each factor, and m is the number of factors. For a three-factor five-level orthogonal experiment, this study chose L_25_ (5^6^) instead of L_25_ (5^3^) for two main reasons. Firstly, L_25_ (5^6^) is the standard orthogonal table recommended statistically, which ensures that each level of each factor appears in the experiment in a balanced manner while maintaining the orthogonality of the design. Secondly, to make an analysis of variance (ANOVA) of the orthogonal experiment results, it is necessary to set error columns. Since the interaction between the two factors is not considered, the remaining three columns (D, E, and F) are used as error columns to estimate the experimental error in ANOVA [33]. When designing orthogonal experiments, it is essential to ensure orthogonality in both the factor columns and the error columns, guaranteeing that each factor in the experiment is independent and does not interfere with others. This requires the combinations of different factors to be evenly distributed throughout the experiment. By doing so, the effect of each factor can be observed independently, ensuring the reliability and accuracy of the experimental results. A total of 25 groups of UCS tests were designed, as shown in Table 5. Each group consisted of three parallel specimens.

#### 2.2.2. Specimen Preparation

In this study, cylindrical specimens of ASC were prepared according to the standard of the American Society of Testing Materials (ASTM C349-18) [34], with dimensions of 50 mm in diameter and 100 mm in height. The preparation of these specimens was carried out using precision-engineered steel molds to ensure uniformity and consistency in their geometric characteristics. The utilization of these molds was critical in maintaining the integrity of the specimens throughout the preparation phase, thereby facilitating accurate and reliable results in subsequent testing procedures.

The preparation of the ASC specimens was conducted with a stringent sequence of material addition. Initially, aeolian sand was introduced into the mixing process, followed by ordinary Portland cement, and, finally, water. The aeolian sand, after precise weighing, was placed into a mixer (NJ-160B, Zhongke Road Construction Instrument Equipment Co., Ltd., Beijing, China). Subsequently, ordinary Portland cement was added following a predetermined dosage, and an initial mixing duration of 30 s was performed. Thereafter, a predetermined mass of water was introduced, and the mixing continued for an additional 5 min to ensure the homogeneity of the mixture.

Before filling the steel molds with the homogenized cement mortar, the interior walls of the molds were evenly coated with Vaseline as a lubricant to facilitate the demolding process. The cement mortar was divided into three equal parts, each of which was sequentially poured into the mold, and compacted 18 times using a compaction device, maintaining a consistent drop height for each compaction. To prevent the formation of stratified structures within the specimens, the surface was lightly roughed with a sharp instrument after compaction. It is crucial to underscore that, during the process of pouring the mortar, it was imperative to ensure that the mass of each sample remained consistent.

Following completion of the filling and compaction, the molds were covered with plastic film to minimize water evaporation. The specimens were cured in an indoor environment for 48 h before demolding. Subsequently, following the Chinese national standard (GB/T 50081-2019) [35], the specimens were placed in a standard curing box maintained at a temperature of 20 ± 2 °C and relative humidity of not less than 95%, until the predetermined curing time was reached [36].

This series of operations ensured the standardization and accuracy of the specimen preparation and testing procedures, providing a reliable foundation for subsequent mechanical performance analysis.

#### 2.2.3. UCS Tests

Upon completion of the designated curing time, the UCS tests were conducted on the specimens to evaluate their mechanical properties. The UCS tests of ASC were conducted on a UCS test machine (YAW-300D, Jinan Hengle Xingke, Jinan, China). The loading rate of the UCS test was a constant rate of 0.8 mm/min. In this study, the UCS values of parallel tests in the same group deviated from the mean by no more than 15%. As a result, the mean value of each group can be used as the evaluation index of the UCS, as listed in Table 6.

#### 2.2.4. Microstructure Test

To enhance the observation of the morphology of hydration products in ASC, this study employed optical microscopy (Zeiss Axio Scope.A1, Carl Zeiss, Oberkochen, Germany) and field emission SEM. Since ASC is a non-conductive material, gold sputtering was performed before SEM observation to enhance the conductivity of the samples and obtain clearer images.

#### 2.2.5. XRD Test

The mineral compositions of ASC were determined by the X-ray diffractometer (Advance D8, Bruker, Karlsruhe, Germany). The test samples were obtained from the ASC specimen after the UCS test. The scanning range was set as 5°~65°.

## 3. Results

### 3.1. UCS of ASC

#### 3.1.1. Range Analysis

The arrangement of factors and levels and the UCS values are listed in Table 6. 

The range analysis of UCS is shown in Table 6. *K_i_* is the sum of UCS at level *i* under each factor, and *κ_i_* is the mean of UCS at level *i* under each factor. *κ_i_* can be employed to ascertain the optimal level under each factor. The level corresponding to the largest *κ_i_* is the optimal level for that factor. Then, combine the optimal levels of each factor to attain the optimal factor-level scheme.

The formula for calculating *K_i_* and *κ_i_* are as shown in Equations (3) and (4):

(3) $$K_{i}=\sum_{j=1}^{n}X_{ij}$$

(4) $$\kappa_{i}=\frac{K_{i}}{n}$$

where *K*_*i*_ is the sum of level *i* of a certain factor, *X*_*ij*_ is represented as the *j*-th value at level *i*, and *n* is the number of level *i* for each factor.

Use UCS values for factor A at level 1 to illustrate the calculation:

$$\begin{array}{l} K_{1}=\;0.12\;+\;0.35\;+\;0.49\;+\;0.80\;+\;1.07\;=\;2.83\\ \kappa_{1}=\frac{2.83}{5}\approx 0.57 \end{array}$$

The order of different factors affecting the test indexes can be obtained by using the range analysis, and the more significant the effect on the test indexes, the greater the range value of the factor [33]. *R* is the difference between max *κ_i_* and min *κ_i_* under each factor, which is used to judge the influence degree of each factor on UCS. The range value *R* is obtained as follows:

(5) $$R=\max\kappa_{i}-\min\kappa_{i}$$

The range analysis result of UCS is *R*_B_ = 1.13 > *R*_A_ = 0.36 > *R*_C_ = 0.27, which indicates that the significance order of UCS is B > A > C (cement dosage > curing time > water dosage).

To find the optimal combination scheme, the *κ_i_* of three influences were compared separately. For curing time, *κ*_5_ > *κ*_4_ > *κ*_3_ > *κ*_2_ > *κ*_1_, for cement dosage *κ*_5_ > *κ*_4_ > *κ*_3_ > *κ*_2_ > *κ*_1_, for water dosage *κ*_3_ > *κ*_4_ > *κ*_2_ > *κ*_5_ > *κ*_1_. Based on this analysis, A_5_B_5_C_3_ (curing time: 90 d; cement dosage: 11%; water dosage: 9%) is the optimum factor level and the optimum level combination.

#### 3.1.2. Analysis of Variance (ANOVA)

ANOVA, commonly known as the *F*-test, can be used in orthogonal experiments to test the significance of factors and separates the influence of factors from experimental error. The fluctuation of data due to changes in the level of factors, errors, etc., cannot be obtained by the range analysis method, which can be supplemented by ANOVA [33]. So, after conducting range analysis on the results of orthogonal experiments, ANOVA is typically performed as well.

The *F*-value, the statistic used in the ANOVA, represents the ratio of the sum of squared deviations to the degrees of freedom within groups. The *F*-value of the *F*-test is calculated as follows:

(6) $$\left\{\begin{array}{l} S^{2}=\sum {\left(X-\bar{X}\right)}^{2}\\ f=k-1\\ f_{e}=n-k\\ V_{J}=S^{2}/f\\ V_{e}=S_{e}/f_{e}\\ F=V_{J}/V_{e} \end{array}\right.$$

where *S*^2^ is the sum of squared deviations, *X* is the experimental data, and $\bar{X}$ is the total mean. *f* is the degree of freedom, *k* is the number of levels of each factor in a column, and *n* is the number of all index values. *V*_J_ denotes the mean square of the and *V*_e_ denotes the mean square of the error columns.

The *F*-test critical value table (*F*_α_) provides *F*-values corresponding to different significance levels. For instance, when α = 0.05, with factor degrees of freedom *f* = 4 and error degrees of freedom *f* = 12, the critical value is *F*_0.05_(4,12) = 3.26. To determine whether there is a significant difference, the calculated *F*-value can be compared with the critical value from the *F*-test table. If the calculated *F*-value is greater than the critical value, it indicates that the factor is significant. A higher *F*-value suggests a stronger significance of the factor.

**Figure 4:**
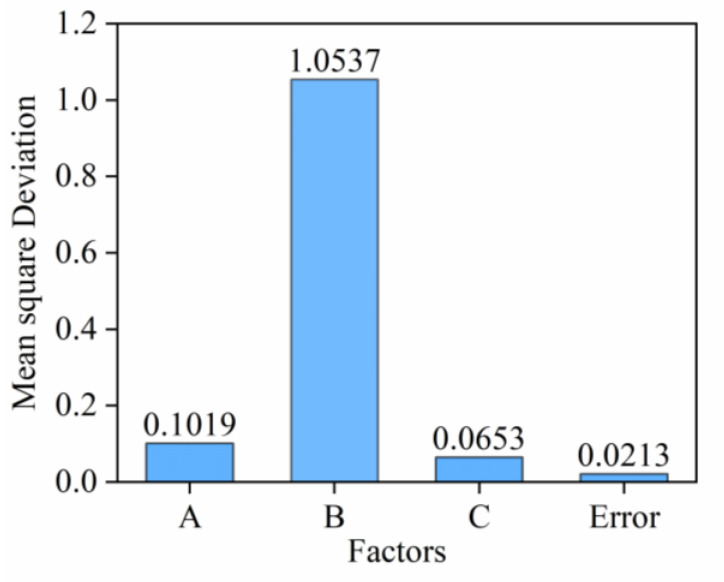
Effect of each factor on the mean squared deviation of the UCS of ASC.

According to the methods of ANOVA mentioned above, the results of ANOVA can be seen in Table 7 and Figure 4. The *F*-values of the three influencing factors A, B, and C are 4.7840, 49.4695, and 3.0657 respectively. When the *F*-value is larger, the change of the factor has a more significant impact on UCS. It can be obtained that the order of significance of each factor on UCS is B > A > C (cement dosage > curing time > water dosage), which is consistent with the range analysis results above.

The rightmost column of Table 7 provides the critical values of *F*_α_ (4,12) for different levels of α in the *F*-test. The *F*-value at α = 0.05 in the standard table is 3.26. The *F*-value of factor C is less than the *F*_0.05_ (4,12) = 3.26, i.e., the change in the UCS of the ASC is not significantly affected by the fluctuation of water dosage when α = 0.05. The *F*-values for factor A and factor B are larger than *F*_0.05_ (4,12) = 3.26, i.e., when α = 0.05, both the A and the B have significant effects on the USC of ASC.

#### 3.1.3. Standard Deviation Analysis

Standard deviation (*SD*) is a crucial statistical measure that quantifies the dispersion of data points around the mean [37]. In this study, the standard deviation for each factor was calculated to assess the variability of the UCS under different factors of A, B, and C.

The formula for *SD* is given by:

(7) $$SD=\sqrt{\frac{1}{N-1}\sum_{i=1}^{N}{\left(x_{i}-\bar{x}\right)}^{2}}$$

where *SD* is the standard deviation, *N* is the number of observations, *x_i_* is the individual observation, and $\bar{x}$ is the mean of the observations. 

The calculated *SD* for each factor at different levels is shown in Table 8.

Factor A (curing time). *SD* ranges from 0.303 to 0.700, indicating that as curing time increases, the variability in strength also increases. Notably, when curing times are at level 4 and level 5, the standard deviations are larger, reflecting higher uncertainty in strength. This may be attributed to the extended curing time allowing for further development and changes in the internal structure of the material.

Factor B (cement dosage). The variability in *SD* for cement dosage is relatively small, with a maximum of 0.380 and a minimum of 0.028. This indicates that strength fluctuates less with different cement dosages, suggesting that changes in cement dosage have a significant impact on strength stability. Overall, the stability of cement dosage outperformed that of the other factors.

Factor C (water dosage). *SD* for water dosage ranges from 0.323 to 0.624, demonstrating a larger variation in strength under different water dosages. Particularly, when water dosages are at level 3 and level 4, *SD* reaches 0.620 and 0.624, respectively, indicating that the choice of water dosage at these levels significantly affects strength, potentially leading to inconsistencies in strength.

#### 3.1.4. Effects of Each Influencing Factor on the UCS of ASC 

**Figure 5:**
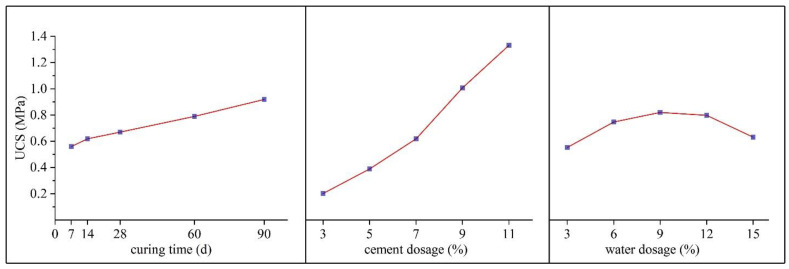
Effect of factors at different levels of UCS.

The effects of the three factors (curing time, cement dosage, and water dosage) on the UCS based on the range analysis are shown in Figure 5.

As shown in Figure 5, the UCS increases with the increase in curing time, but the increase rate of UCS slows down when the curing time is 14 d because the hydration reaction of cement in ASC starts rapidly in the early stages and then gradually slows down as the reaction progresses.

Figure 5 shows that the UCS of ASC exhibits a positive correlation with the increase in cement dosage. Specifically, at a cement dosage of 3%, the UCS of ASC is 0.20 MPa, when the cement dosage rises to 11%, the UCS increases to 1.33 MPa. The rate of UCS increase in ASC is relatively slow when the cement dosage ranges from 3% to 7%. However, beyond a cement dosage of 7%, the rate at which the UCS of ASC increases with cement dosage accelerates significantly. This acceleration is likely attributable to the more extensive hydration reactions that occur with higher cement dosages, leading to the formation of a greater quantity of hydration production such as calcium silicate hydrate (C-S-H) gel, which is the primary contributor to the strength of ASC [38].

In contrast to factors A and B, the UCS of ASC does not increase with the water dosage. Instead, it exhibits a trend of increasing first and then decreasing as the water dosage increases. As can be seen in Figure 5, the UCS reaches a maximum value at the water dosage of 9%, with a recorded UCS of 0.83 MPa. This indicates that the optimal water dosage is 9%. Beyond this level, excessive water may lead to a dilution effect, and the excess water evaporates after hydration, resulting in additional voids, thereby reducing the strength of ASC.

### 3.2. Microstructure and Mineral Composition of ASC

Optical microscopy, SEM, and XRD were used in the microscopic analysis of ASC.

#### 3.2.1. Microstructure of ASC

**Figure 6:**
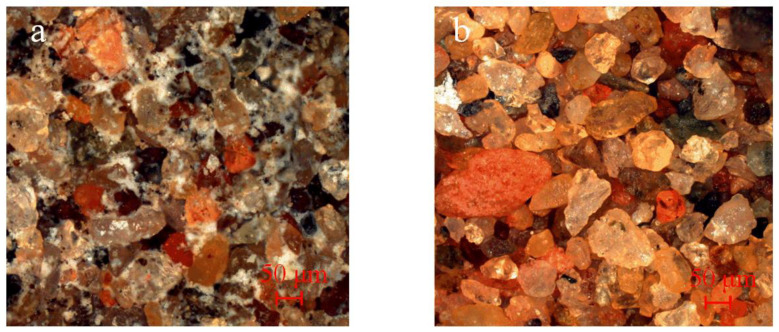
Optical microscopy images (magnified 50×): (**a**) ASC and (**b**) original aeolian sand.

As shown in Figure 6b, the surface of the sand particles is smooth, with a variety of colors and large pores, while ASC contains hydration products among the sand particles that are wrapped around and cement the particles together.

**Figure 7:**
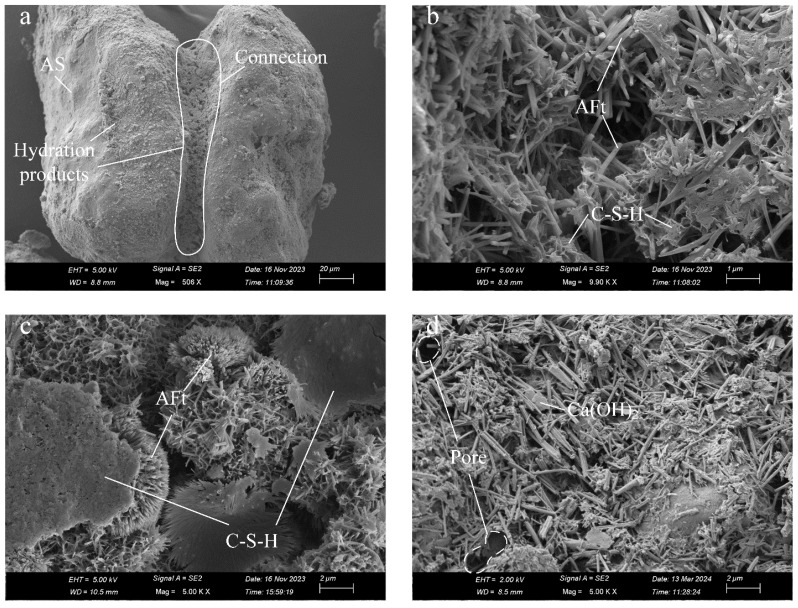
SEM micrographs of ASC magnified: (**a**) 506×; (**b**) 9900×; (**c**) 5000×; (**d**) 5000×.

As illustrated in Figure 7a, the microscopic morphology shows two aeolian sand particles wrapped together by the hydration products of cement. Figure 7a also reveals an aeolian sand particle surface that has not participated in the hydration reaction. In Figure 7b–d, it is observed that the hydration products mainly consist of flocculent and platy C-S-H, flake calcium hydroxide (CH), and prismatic and clustered needle-like ettringite crystals (AFt), a common product of the hydration reaction in Portland cement, which is formed due to free aluminum [39], with pores visible within these products. The microstructural morphology of the hydration products in ASC is consistent with results reported for pure cement hydration products [28,40].

**Figure 8:**
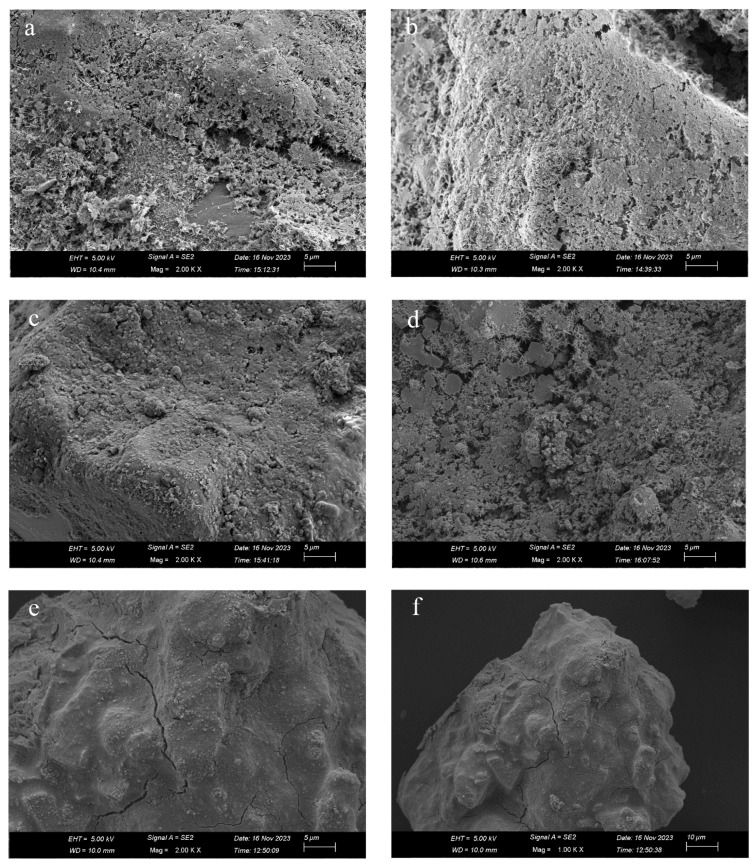
SEM micrographs of ASC with the same curing time of 14 d but different cement dosages: (**a**) 3%; (**b**) 5%; (**c**) 7%; (**d**) 9%; (**e**) 11%; (**f**) 11%.

Figure 8 shows SEM images of Samples 6–10 with different cement dosages but the same curing time of 14 d. Figure 8a shows an SEM image of ASC with a cement dosage of 3%. It can be observed that the hydration products are not only less in quantity but also relatively sparse. There is a significant area on the surface of aeolian sand that is not covered by hydration products. Figure 8b shows an SEM image of ASC with a cement dosage of 5%. The hydration products produced on the sand particle surfaces increase in quantity and become more compact. Microscopic images of ASC with cement dosages of 7%, 9%, and 11% are also presented in Figure 8. When compared with the low cement dosage treatment (3% and 5%), there are obvious changes. Specifically, hydration products on the surface of aeolian sand are tightly integrated into the entire structure. Especially, in Figure 8e,f, the aeolian sand particles are evenly coated, forming a shell, and microcracks can be seen, with a size of about 50 nm. These microcracks in Figure 8e–f are caused by the growth of crystals in the hydration products, which increases the internal expansion stress. Lee [41] found that the expansion of ettringite causes cracks on the surface of the hydration product. They are different to the pores in Figure 8a–d, which cannot bond together due to insufficient amounts of hydration products. 

**Figure 9:**
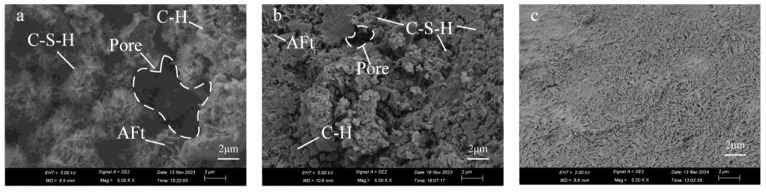
SEM micrographs of ASC with the same cement dosage of 9% but different curing times: (**a**) 7 d; (**b**) 14 d; (**c**) 90 d.

Figure 9 shows the SEM images of samples (test IDs 4, 9, 24) with different curing times but the same cement dosage of 9%. Figure 9a shows an SEM image of ASC with a curing time of 7 d. The hydration products are less and loosely distributed, and there are large pores in the hydration products. The hydration products are mainly needle-shaped C-S-H, prismatic AFt, and flake-shaped CH. Figure 9b shows an SEM image of ASC with a curing time of 14 d. Compared with ASC with a curing time of 7 d, the hydration products fill the pores and cover the surface of aeolian sand. The shape changes from needle-like (considered to be the very early age morphology of C-S-H [42,43]) to flocculated and plate-like. This transformation reflects the evolution of its microstructure and the enhancement of its mechanical properties. The prismatic crystals of AFt increase in diameter and the abundance of CH escalates significantly, manifesting a conspicuous plate-like configuration. As shown in Figure 9c, for ASC with a curing time of 90 days, a confluence of various hydration products has amalgamated to form a dense plate-like shell, completely enveloping the surface of the aeolian sand.

Based on microstructural analysis, the hydration products of ASC develop on the surface of sand particles. As the hydration products increase and interconnect, they occupy interstitial voids among the aeolian sand particles, fostering cohesion between adjacent particles. As the contact area between particles increases, the mechanical properties of ASC also exhibit augmentation.

#### 3.2.2. XRD Patterns

**Figure 10:**
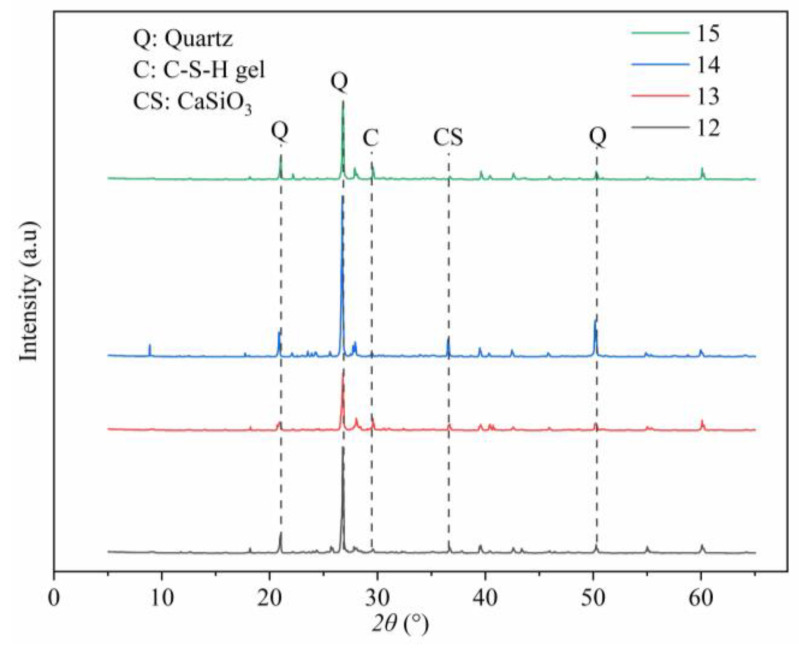
XRD patterns of ASC samples with test IDs from 12 to 15.

Figure 10 shows the XRD patterns of ASC samples (test IDs from 12 to 15), with the same curing time of 14 d. Cement and water dosages are mentioned in Table 5.

By observing the peaks of the XRD patterns, the most possible compounds generated in the samples are C-S-H and calcium aluminum silicate hydrate (C-A-S-H), the typical hydration products of cement. In general, the diffraction peak of C-S-H gel is near 2*θ* = 29° [44]. As shown in Figure 10, a dispersion peak is observed at approximately 2*θ* = 29.5°, which has been identified as the characteristic peak of C-S-H based on the PDF standard cards. The XRD patterns of Samples 12, 13, and 15 exhibit a progressive enhancement in the intensity of the C-S-H peaks, with the cement dosage increased. This observation, consistent with established scientific principles, indicates that the quantity of C-S-H produced during hydration is positively correlated with the increment of cement dosage.

In the hydration reaction, water also plays a role. This research, through the analysis of XRD patterns of both the reactants and products, elucidates that insufficient water supply within the reaction system hinders the hydration process. On the one hand, this perspective can be explained by considering the products of the hydration reaction. It can be observed that the intensity of the C-S-H peak in the XRD pattern of Sample 14 is significantly lower than that of the other three samples, which contradicts contrasts with the trends observed in the C-S-H characteristic peaks from Samples 12, 13, and 15. This discrepancy may be attributed to the extremely limited water dosage in Sample 14, which is only 3% in this study. Low water dosage likely restricts the hydration reaction, resulting in fewer hydration products and a noticeably lower intensity of the C-S-H peak. On the other hand, this perspective can be analyzed from the standpoint of reactants in hydration reactions. As shown in Table 5, the cement dosage in Sample 14 is lower than that in Sample 15, being 9% and 11%, respectively, while the mass of aeolian sand in both samples is equal. By comparing the XRD patterns of Sample 15 and Sample 14, it is evident that the peaks of quartz and calcium silicate (one of the main components of cement) in the Sample 14 pattern are indeed higher. This indicates that a lower proportion of the cement in Sample 14 has undergone hydration and formed hydration products. So, there is a higher proportion of unreacted cement in Sample 14. Based on the XRD patterns, it is clear that insufficient water can inhibit the hydration reaction both in terms of reactant and product quantities.

However, in the XRD pattern of Sample 14, despite the reduced peak intensity of C-S-H gel compared with Samples 12 and 13, there is no corresponding decline in the macroscopic mechanical performance. This observation can be attributed to the presence of multiple distinct peaks in the ranges from 2*θ* = 26° to 2*θ* = 29° in the XRD pattern of Sample 14, corresponding to the characteristic peaks of calcium aluminate silicate hydrate (C-A-S-H) [45]. As a critical hydration product, C-A-S-H also has been shown to have a significant positive impact on macroscopic mechanical performance. Therefore, despite the lower peak intensity of C-S-H in Sample 14, the presence of C-A-S-H effectively compensates for this deficiency, maintaining and even improving the macroscopic mechanical performance of the sample.

Furthermore, according to the PDF standard cards, the XRD pattern of Sample 14 shows a characteristic peak of calcium carbonate (Ca(CO)_3_) at approximately 2*θ* = 8°, which may be due to the reaction between calcium hydroxide (Ca(OH)_2_) generated during hydration and carbon dioxide (CO_2_) in the environment. This reaction may further influence the sample’s microstructure and macroscopic performance.

### 3.3. Correlation between Microstructure, Mineral Composition, and Mechanical Performance

Based on the microscopic investigation, the strength of ASC primarily arises from two main aspects. Firstly, the hydration of cement within the aeolian sand matrix fills and seals interstitial voids, densifying the overall structure. Secondly, the cementitious hydration products effectively bond individual aeolian sand particles into a cohesive aggregate, significantly enhancing the UCS.

Cement is the primary reactant in hydration reactions, directly determining the maximum quantity of hydration products generated. Increasing cement dosage provides more reactants, which then produce greater quantities of hydration products. With prolonged curing time, hydration reactions proceed for a longer duration, resulting in increased hydration product formation. Although water is essential for the hydration reaction, in most cases, the existing amount of water is sufficient to support a complete reaction. Additional water does not only significantly increase the quantity of hydration products but even results in additional voids, thereby reducing strength. This clearly explains the order of influential significance of the three factors on UCS as cement dosage > curing time > water dosage. It also elucidates the impact trends of these three factors on UCS, as mentioned above.

## 4. Discussion

### 4.1. Key Findings of This Study

This study systematically evaluated the effects of curing time, cement dosage, and water dosage on the UCS of ASC, resulting in several key findings as follows:

(1) Effect of curing time. Prolonging the curing time significantly increased the UCS of ASC. Specifically, UCS increased substantially at curing times of 60 and 90 days, indicating the importance of long-term curing for ASC performance.
(2) Effect of cement dosage. With increasing cement dosage, the UCS of ASC improved significantly. Higher cement dosages provided more binder, filling the voids between sand particles and enhancing the overall strength and stability of the material.
(3) The UCS of ASC reached its maximum at a water dosage of 9% and then decreased with further increases in water dosage, indicating that there was an optimal dosage for the strength. These research findings aligned well with existing studies [46,47,48].

Through microstructural analysis, we observed that a higher cement content and moderate water content led to the formation of more hydration products. Over time, these products became denser, filling the pores in the material and thereby increasing its strength and density [49]. This finding further supports the results of macroscopic mechanical performance tests.

### 4.2. Influence of Water/Cement Ratio (w/c)

Although the orthogonal experimental design in this study did not specifically include the water/cement ratio (*w*/*c*) as a variable, the results and the existing literature indicate that *w*/*c* plays a crucial role in determining the UCS of cement-stabilized materials [46,49]. SEM images and XRD patterns showed that adequate water promoted sufficient hydration reactions, but excessive water increased porosity, weakening the structural integrity of the material. This strongly demonstrated that the *w*/*c* significantly affected the strength of ASC.

The *w*/*c* of the optimal combination in this study was 0.82, which was higher than the optimal *w*/*c* for conventional sand. Several factors contributed to this phenomenon. Firstly, the significantly smaller particle size of aeolian sand resulted in a larger specific surface area. This increased surface area required relatively more water to ensure adequate hydration and coating of the particles. The high surface area created a higher demand for water to cover the additional surface area and facilitate the necessary chemical reactions for cement hydration. Secondly, the natural moisture content of aeolian sand was typically lower compared with that of conventional sand. Lower initial water content required more additional water to reach the same hydration state.

By acknowledging the influence of *w*/*c* on UCS, even though it was not a primary variable in this study, we can suggest that future research should include *w*/*c* as a critical parameter. This would help in better understanding and optimizing the mechanical properties of ASC.

### 4.3. Standard Deviation Analysis

In this study, both range analysis and ANOVA were used to assess the influence of curing time, cement dosage, and water dosage on the UCS of ASC. The results from these analyses were consistent and provided a clear ranking of the factors based on their impact on UCS.

From the range analysis, *R*_A_ = 0.36, *R*_B_ = 1.13, and *R*_C_ = 0.27, indicating that cement dosage had the most significant influence on UCS, followed by curing time and water dosage. From ANOVA, *F*_A_ = 4.7840 (significant), *F*_B_ = 49.4695 (significant), and *F*_C_ = 3.0657 (not significant), confirming that cement dosage was the most significant factor affecting UCS, followed by curing time, while the impact of water dosage was not statistically significant. From the SD analysis, *SD*_A_ ranged from 0.303 to 0.700, indicating considerable variability. *SD*_B_ ranged from 0.028 to 0.380, showing relatively low variability. *SD*_C_ ranged from 0.323 to 0.624, indicating significant variability.

The consistency between range analysis and ANOVA results reinforced the conclusion that cement dosage was the primary factor influencing UCS, with curing time also playing a significant role. The SD analysis highlighted the data’s stability and consistency. Cement dosage showed a significant impact with low variability, while curing time and water dosage exhibited higher variability. This could be explained by the variations in the mixing and compaction processes, which could result in a non-uniform distribution of cement and water within the sand matrix. Such non-uniformity could cause differences in the strength development of the material, thereby contributing to higher variability in UCS.

## 5. Conclusions

This study analyzed the influence of curing time, cement dosage, and water dosage on the UCS of ASC. The research compared the degree of influence of these three factors on the mechanical properties of ASC and determined the optimal combination design. Furthermore, it explained the microscopic mechanism of cement stabilization of aeolian sand. The conclusions were as follows:

(1) Through orthogonal experiments, the optimal combination design was obtained (curing time: 90 d; cement dosage: 11%; water dosage: 9%). Among the three factors, cement dosage had the most significant impact on the UCS of ASC, followed by curing time, and water dosage was relatively the least significant.
(2) Effect of curing time. The UCS of ASC increased with the prolongation of curing time due to the gradual progress of the hydration reaction of cement, which generated more hydration products, reinforcing the inter-particle bonding in the aeolian sand–cement composite. Notably, UCS increased substantially at curing times of 60 and 90 days, indicating the importance of long-term curing for the performance of ASC.
(3) Effect of cement dosage. The UCS of ASC increased with the increase in cement dosage due to the increased amount of hydration products generated by the hydration reaction of cement, enhancing the inter-particle bonding force of aeolian sand and leading to the infill of interstitial voids, thereby densifying the overall structure.
(4) Effect of water dosage. The UCS of ASC reached its maximum at a water dosage of 9%, then decreased with further increases in water dosage. An optimal amount of water promoted adequate hydration reactions, while excessive water increased porosity and weakened the structural integrity of the material. Optimizing water dosage was crucial for enhancing the mechanical properties of ASC.
(5) Microstructural analysis revealed that longer curing time, higher cement dosages, and moderate water dosages generated more hydration products, filling the voids in the ASC and enhancing its strength and density. These microstructural changes further supported the results of the macro-mechanical tests.

In conclusion, this study provides important insights into the stabilization mechanism of ASC through detailed experiments and analyses and offers practical recommendations for engineering applications. These findings will contribute to improving the construction standards of infrastructure in desert areas and promoting the development of this field.

## Figures and Tables

**Table 1:** Physical properties of raw materials.

Parameters	Aeolian Sand	Portland Cement
Color	Yellow	Grey
Moisture content	0.54%	-
Natural density	1.38 g/cm^3^	2.62 g/cm^3^
Natural porosity	52%	-
Particle size distribution		
*d*_10_ ^a^	131.95 μm	5.24 μm
*d*_30_	210.22 μm	14.51 μm
*d*_60_	345.58 μm	41.12 μm
*C*_c_ ^b^	0.97	0.98
*C*_u_ ^c^	2.62	7.85

Notes: ^a^ *d*_x_ represents the *x*% volume percentage of particles with a diameter less than *d*_x_ mm. ^b^ coefficient of curvature. (*C*_c_ = *d*_30_^2^/*d*_10_*d*_60_). ^c^ coefficient of uniformity (*C*_u_ = *d*_60_/*d*_10_).

**Table 2:** Main chemical compositions of raw materials.

Materials	Chemical Composition (%)												
	SiO_2_	CaO	Fe_2_O_3_	Al_2_O_3_	MgO	SO_3_	Na_2_O	K_2_O	Cr_2_O_3_	TiO_2_	P_2_O_5_	MnO	Others
Aeolian sand	69.78	6.61	1.96	7.89	1.07	1.51	2.05	1.86	0.33	0.39	0.06	0.04	6.45
Portland cement	26.63	46.00	3.89	10.59	3.66	2.68	1.19	1.13	0.64	0.50	0.18	0.17	2.74

**Table 3:** Mineralogical composition of aeolian sand.

Composition	Quartz	Felspar	Calcite	Mica	Kaolinite	Else
Content (%)	76.2	6.9	7.2	3.0	2.3	4.4

**Table 4:** Orthogonal factors and levels.

Factors	Levels				
	1	2	3	4	5
A: Curing time (d)	7	14	28	60	90
B: Cement dosage (%)	3	5	7	11	13
C: Water dosage (%)	3	6	9	12	15

**Table 5:** L_25_ (5^6^) orthogonal design table.

Test ID	A	B	C	D	E	F
1	1 (7 d)	1 (3%)	1 (3%)	1	1	1
2	1	2 (5%)	2 (6%)	2	2	2
3	1	3 (7%)	3 (9%)	3	3	3
4	1	4 (9%)	4 (12%)	4	4	4
5	1	5 (11%)	5 (15%)	5	5	5
6	2 (14 d)	1	2	3	4	5
7	2	2	3	4	5	1
8	2	3	4	5	1	2
9	2	4	5	1	2	3
10	2	5	1	2	3	4
11	3 (28 d)	1	3	5	2	4
12	3	2	4	1	3	5
13	3	3	5	2	4	1
14	3	4	1	3	5	2
15	3	5	2	4	1	3
16	4 (60 d)	1	4	2	5	3
17	4	2	5	3	1	4
18	4	3	1	4	2	5
19	4	4	2	5	3	1
20	4	5	3	1	4	2
21	5 (90 d)	1	5	4	3	2
22	5	2	1	5	4	3
23	5	3	2	1	5	4
24	5	4	3	2	1	5
25	5	5	4	3	2	1

**Table 6:** Range analysis of UCS values of ASC.

Test ID	A	B	C	D	E	F	UCS (MPa)
1	1	1	1	1	1	1	0.12
2	1	2	2	2	2	2	0.35
3	1	3	3	3	3	3	0.49
4	1	4	4	4	4	4	0.80
5	1	5	5	5	5	5	1.07
6	2	1	2	3	4	5	0.22
7	2	2	3	4	5	1	0.41
8	2	3	4	5	1	2	0.65
9	2	4	5	1	2	3	0.89
10	2	5	1	2	3	4	0.92
11	3	1	3	5	2	4	0.24
12	3	2	4	1	3	5	0.42
13	3	3	5	2	4	1	0.64
14	3	4	1	3	5	2	0.81
15	3	5	2	4	1	3	1.24
16	4	1	4	2	5	3	0.26
17	4	2	5	3	1	4	0.40
18	4	3	1	4	2	5	0.54
19	4	4	2	5	3	1	1.15
20	4	5	3	1	4	2	1.58
21	5	1	5	4	3	2	0.18
22	5	2	1	5	4	3	0.38
23	5	3	2	1	5	4	0.79
24	5	4	3	2	1	5	1.41
25	5	5	4	3	2	1	1.85
*K*_1_	2.83	1.02	2.77	3.80	3.82	4.17	
*K*_2_	3.09	1.96	3.75	3.58	3.87	3.57	
*K*_3_	3.35	3.11	4.13	3.77	3.16	3.26	
*K*_4_	3.93	5.06	3.98	3.17	3.62	3.15	
*K*_5_	4.61	6.66	3.18	3.49	3.34	3.66	
*κ*_1_	0.57	0.20	0.55	0.76	0.76	0.83	
*κ*_2_	0.62	0.39	0.75	0.72	0.77	0.71	
*κ*_3_	0.67	0.62	0.83	0.75	0.63	0.65	
*κ*_4_	0.79	1.01	0.80	0.63	0.72	0.63	
*κ*_5_	0.92	1.33	0.64	0.70	0.67	0.73	
*R*	0.36	1.13	0.27	0.13	0.14	0.20	
Degree of influence	2	1	3	6	5	4	
Optimal factor-level scheme: A_5_B_5_C_3_ (curing time: 90 d; cement dosage: 11%; water dosage: 9%).							

**Table 7:** Variance analysis of UCS values of ASC.

Source	*SS*	*df*	*MS*	*F*	*F*_α_(4,12)
A	0.4075	4.0000	0.1019	4.7840	*F*_0.01_(4,12) = 5.41 *F*_0.05_(4,12) = 3.26 *F*_0.10_(4,12) = 2.48
B	4.2148	4.0000	1.0537	49.4695	
C	0.2612	4.0000	0.0653	3.0657	
Errors (D, E, F)	0.2550	12.0000	0.0213		

Note: *SS*—sum of squares; *df*—degrees of freedom; *MS*—mean squared deviation; *F*—*F*-Value.

**Table 8:** Standard deviation analysis of UCS values of ASC.

Levels	A	B	C
1	0.374	0.055	0.323
2	0.303	0.028	0.459
3	0.385	0.116	0.620
4	0.559	0.264	0.624
5	0.700	0.380	0.359

## Data Availability

The raw data supporting the conclusions of this article will be made available by the authors on request.
